# Cervical cancer detection in pap smear whole slide images using convNet with transfer learning and progressive resizing

**DOI:** 10.7717/peerj-cs.348

**Published:** 2021-02-18

**Authors:** Anant R. Bhatt, Amit Ganatra, Ketan Kotecha

**Affiliations:** 1 Centre of Excellence for Artificial Intelligence, Military College of Telecommunication Engineering, Mhow, Madhya Pradesh, India; 2 Devang Patel Institute of Advance Technology and Research, Charotar University of Science and Technology, Changa, Gujarat, India; 3 Symbiosis Centre for Applied Artificial Intelligence, Symbiosis International (Deemed University), Pune, Maharashtra, India

**Keywords:** Cervical cytology, Cervical cancer, Transfer learning, Papanicolaou smear, Progressive resizing, Convolution neural network, Sipakmed, Herlev, Metamorphic analysis, Deep learning

## Abstract

Cervical intraepithelial neoplasia (CIN) and cervical cancer are major health problems faced by women worldwide. The conventional Papanicolaou (Pap) smear analysis is an effective method to diagnose cervical pre-malignant and malignant conditions by analyzing swab images. Various computer vision techniques can be explored to identify potential precancerous and cancerous lesions by analyzing the Pap smear image. The majority of existing work cover binary classification approaches using various classifiers and Convolution Neural Networks. However, they suffer from inherent challenges for minute feature extraction and precise classification. We propose a novel methodology to carry out the multiclass classification of cervical cells from Whole Slide Images (WSI) with optimum feature extraction. The actualization of Conv Net with Transfer Learning technique substantiates meaningful Metamorphic Diagnosis of neoplastic and pre-neoplastic lesions. As the Progressive Resizing technique (an advanced method for training ConvNet) incorporates prior knowledge of the feature hierarchy and can reuse old computations while learning new ones, the model can carry forward the extracted morphological cell features to subsequent Neural Network layers iteratively for elusive learning. The Progressive Resizing technique superimposition in consultation with the Transfer Learning technique while training the Conv Net models has shown a substantial performance increase. The proposed binary and multiclass classification methodology succored in achieving benchmark scores on the Herlev Dataset. We achieved singular multiclass classification scores for WSI images of the SIPaKMed dataset, that is, accuracy (99.70%), precision (99.70%), recall (99.72%), F-Beta (99.63%), and Kappa scores (99.31%), which supersede the scores obtained through principal methodologies. GradCam based feature interpretation extends enhanced assimilation of the generated results, highlighting the pre-malignant and malignant lesions by visual localization in the images.

## Introduction

The global cancer burden is estimated to have risen to 18.1 million new cases and 9.6 million deaths in 2018. One in five men and one in six women worldwide develop cancer during their lifetime, and one in eight men and one in 11 women die from the disease. Explicitly, Cervical cancer is the fourth most common malignant tumor threatening women’s health, especially in developing countries. The total number of cervical cancer cases in 2018 reported by the World Health Organization (WHO) was 570,000, and the number of deaths equal to 311,000 cervical cancer ranks fourth for both incidence (6.6%) and mortality (7.5%) (World Health Organization, 2018b). Cervical cancer develops in a woman’s cervix (the uterus’s entrance from the vagina). Human papillomaviruses (HPV)—a ubiquitous virus infection—is a primary cause of cervical cancer in 99% of the cases. Persistent infection of HPV leads to develop cervical cancer in women progressively. Uncontrolled growth of cells caused due to antiapoptotic mutations results in a lump of mass referred to as a tumor, and tumor buds can spread infections to other body parts, leading to severe medical conditions. The mortality and morbidity rate remain high if not detected/cured in due time (World Health Organization, 2018a).

Studies suggest that cervical cancer can be treated successfully if the precancerous lesions are detected in time during cytological screening and Human Papilloma Virus (HPV) test (Saslow et al., 2012). HPV vaccination and detection/treatment are prevention methods in practice. Cervical cancer can be evicted by initiating proactive measures to prevent, carry out regular screening tests and treatment. The conventional Papanicolaou test (Papanicolaou & Traut, 1941), also called the Pap smear test, is an important stage for mitigating the rising challenge of cervical cancer.

A skilled pathologist identifies carcinoma signatures by analyzing morphological features of the cells in microscopic slides manually. Since the manual analysis is subjective to the expert’s knowledge of the disease’s etiology and experience, it may lead to many true negative or false-positive results, leading to incorrect diagnoses and treatments. Also, screening tests carried out in mass imply a higher turn-around for results and substandard screening analysis. The non-availability of expert pathologists and suitable infrastructure restricts cervical cancer screening drives in developing countries.

Deep Learning techniques provide a prodigious prospect for enhanced interpretation and analysis of the pap smear images during Metamorphic Diagnosis of Neoplastic and pre-neoplastic lesions. Since the cells’ morphology undergoes distinct changes during infections, they play a decisive role in identifying carcinoma signatures in pap smear image. Hence, Deep Learning techniques can extract relevant morphology features and carry out Whole Slide Image analysis to identify carcinoma conditions. We followed the Bethesda System (TBS) (Solomon et al., 2002), which explains the cytological classification based on standard medical terms to describe abnormal findings from CPS and LBC. Although the Convolution Neural Networks (CNN) can extract, identify, and classify the carcinoma cells, we elaborate on a novel methodology to improve morphological features’ extraction, thereby increasing accuracy, sensitivity, specificity, and *F*1 scores. State of the art implementation of various CNNs with transfer learning and progressive resizing techniques present a definitive approach for enhanced learning and effective classifications of carcinoma cells.

We studied current implementations/methods on the cervical cancer datasets. We analyzed the datasets and reviewed the recent work, that is, K-Means Clustering, SBRG algorithm (Isa, 2005). It detects the edges of multiple cells/the region of interest (ROI). In addition to the edge detection method, we explored different supervised and unsupervised techniques (Plissiti, Nikou & Charchanti, 2010; Plissiti et al., 2018). In these approaches, detection of nuclei locations was carried out, followed by a refinement step which used this nuclei information (circumference of the nuclei) was performed. Few of them were followed by a classification algorithm to detect the abnormal cells in the images. Both supervised and unsupervised techniques, that is, Support Vector Machines and fuzzy logic, were applied. We also studied various classifiers, that is, K-Nearest Neighbour, Stochastic Gradient Descent, Support Vector Machines (Cortes & Vapnik, 1995), and Random Forest. One of the approaches, that is, DeepPap (Zhang et al., 2017), was also studied in detail. In DeepPap, classification was performed on cropped images (single-cell cropping was carried out considering nuclei as the centroid). However, these methodologies suffer from prominent challenges as follows:-

1. It depends on the localization of nuclei;
2. Detection and classification of images of a single cell only;
3. Comparatively substandard feature extraction for unclear/blurred visual exposure of overlapping cells;
4. Single-cell classification implies Binary classification.

We propose a methodology to subdue the existing binary classification scores and propose a multi-class classification for single-cell and Whole Slide Images of cervical cancer datasets, following the Bethesda System. The proposed approach of superimposing Progressive Resizing with Transfer Learning on CNN yields promising multi-class classification scores with improved visual inferences. Deep learning models are known to have limited interpretability, and it is a challenging and active area of research (Simonyan, Vedaldi & Zisserman, 2013). To enhance the interpretability, we implemented a complementary interpretation with intuitive saliency maps (GradCam) Visualisation. GradCam aids in apprehending the features learned by our model to ensure a high level of transparency in visual interpretation (Zhang, Nian Wu & Zhu, 2018).

We carried out experiments on two different datasets obtained from different Institutes/University. To summarize, our contributions are as follows:

1. To the best of our knowledge, the proposed work is the first implementation to present a singular multi-class classification methodology for Neo-plastic and Pre-Neoplastic lesion detection in Whole Slide Images.
2. Our experiments achieved State-of-the-Art performance scores for binary and multi-class classification on Herlev and SIPaKMeD datasets.
3. The CNN implementation with Progressive Resizing and Transfer Learning techniques yields significant improvements and generated benchmark scores for multi-class classification for whole slide images of the SIPaKMeD dataset, which helps in carrying out metamorphic analysis even for overlapping cell lesions.
4. The article also brings out the comparative study of the results produced by various classifiers and advanced CNN models (trained using Transfer Learning and Progressive resizing techniques) on Herlev and SIPaKMeD datasets.
5. GradCam based feature interpretation extends enhanced assimilation of the generated results, highlighting the pre-malignant and malignant lesions by visual localization in the images.

## Datasets

**Figure 1:**
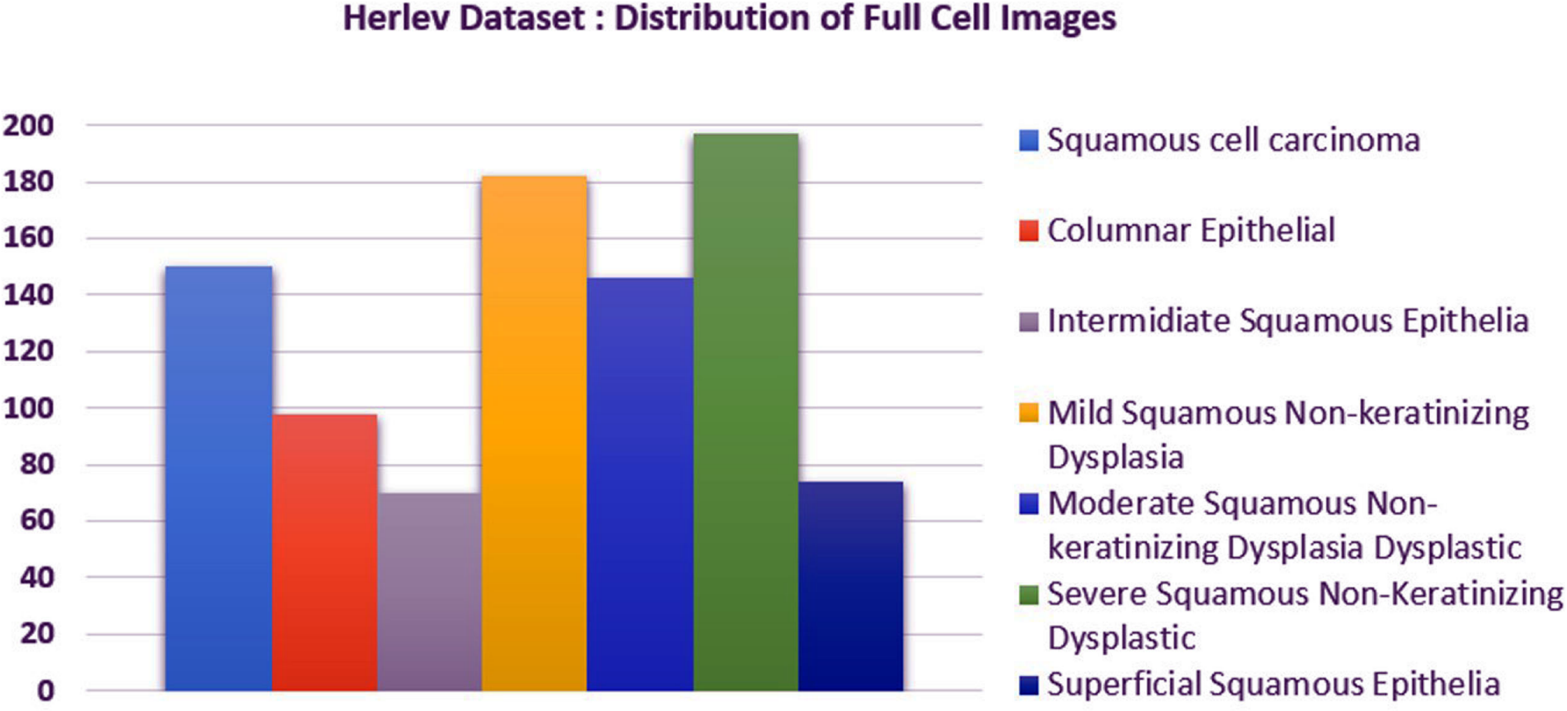
The Herlev Cervical Cancer dataset distribution, categorized into seven classes.

**Figure 2:**
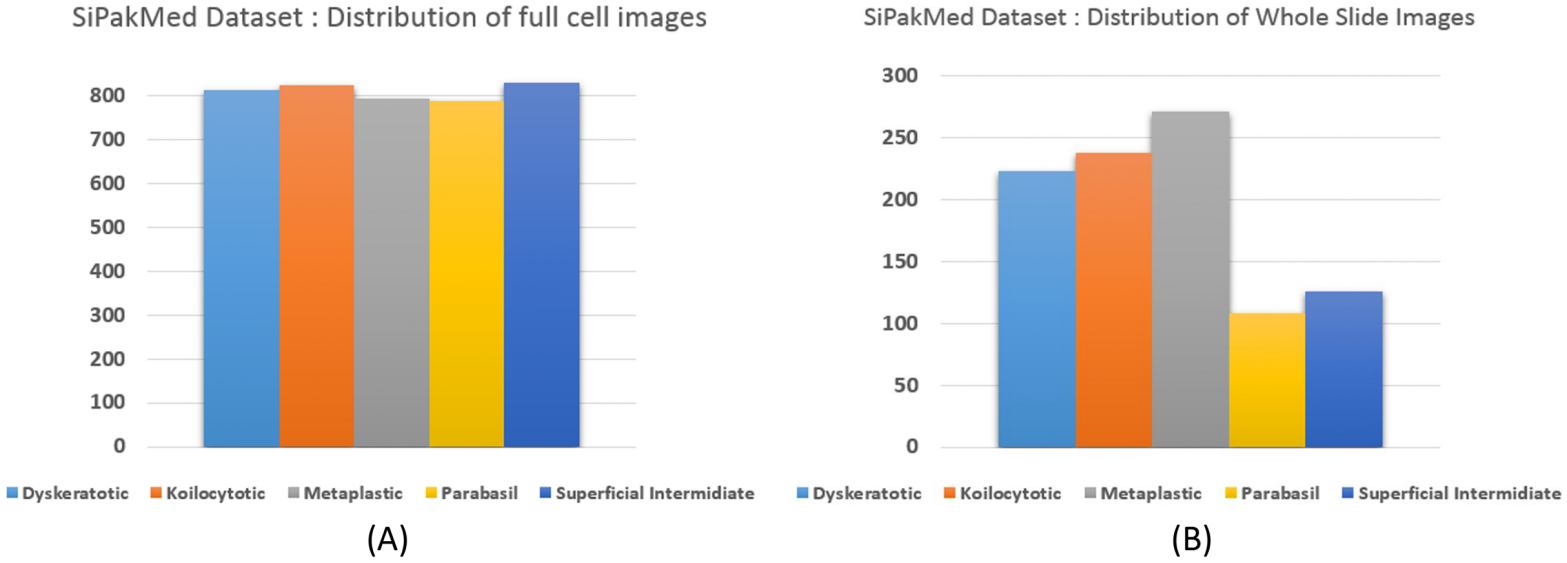
The SIPaKMeD Cervical Cancer dataset distribution, categorized into five classes. The distribution of full cell images at (A) and whole slide image in the datasets at (B) are shown.

**Figure 3:**
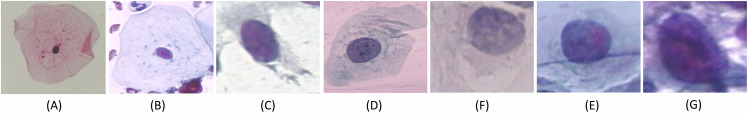
Single cell Images from the Herlev Dataset, categorized into seven classes and shown as (A) superficial squamous epithelia, (B) intermediate squamous epithelia, (C) columnar epithelial, (D) mild squamous non-keratinizing dysplasia, (E) moderate squamous non-keratinizing dysplasia, (F) Severe squamous non-keratinizing dysplasia, (G) squamous cell carcinoma in situ.

**Figure 4:**
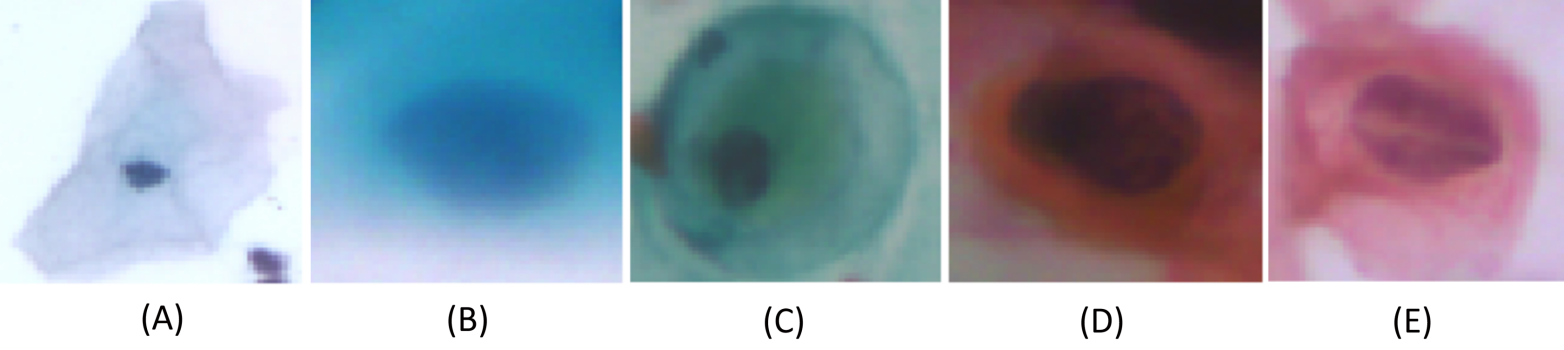
Single-cell images from the SIPaKMeD Dataset, categorized into five classes: (A) Superficial-Intermediate cells, (B) Parabasal cells, (C) Metaplastic cells, (D) Dyskeratotic cells, and (E) Koilocytotic cells.

We carried out experiments on Herlev and SIPaKMeD (Plissiti et al., 2018) databases separately. Herlev database-created at the Herlev University Hospital, Denmark, utilizing a digital camera microscope and contains 917 images of single cells. Skilled cyto-technicians and doctors have annotated each cell into one of seven classes (i.e., Superficial squamous epithelia, Intermediate squamous epithelia, Columnar epithelial, Mild squamous non-keratinizing dysplasia, Moderate squamous non-keratinizing dysplasia, Severe squamous non-keratinizing dysplasia, and Squamous cell carcinoma). The morphological features (i.e., cell shape, nucleus size, nucleus to cytoplasm ratio, nucleus opacities, nucleus dying intensities, cytoplasm opacities, and cytoplasm dying intensities) help differentiate the cells. The SIPaKMed database consists of 4,049 annotated images that have been manually cropped from 966 cluster cell images by the expert cytopathologists into five categories. The cells are classified as normal cells under two types (Superficial-intermediate, Parabasal), as abnormal cells are classified into two categories (Koilocytotic and Dyskeratotic), and as benign categorization (metaplastic) cells. The dataset distributions for Herlev and SIPaKMeD datasets are shown in Figs. 1 and 2, respectively. The specimen pap smear images of the Herlev dataset and SIPaKMeD dataset are shown in Figs. 3 and 4.

## Proposed Methodology

**Figure 5:**
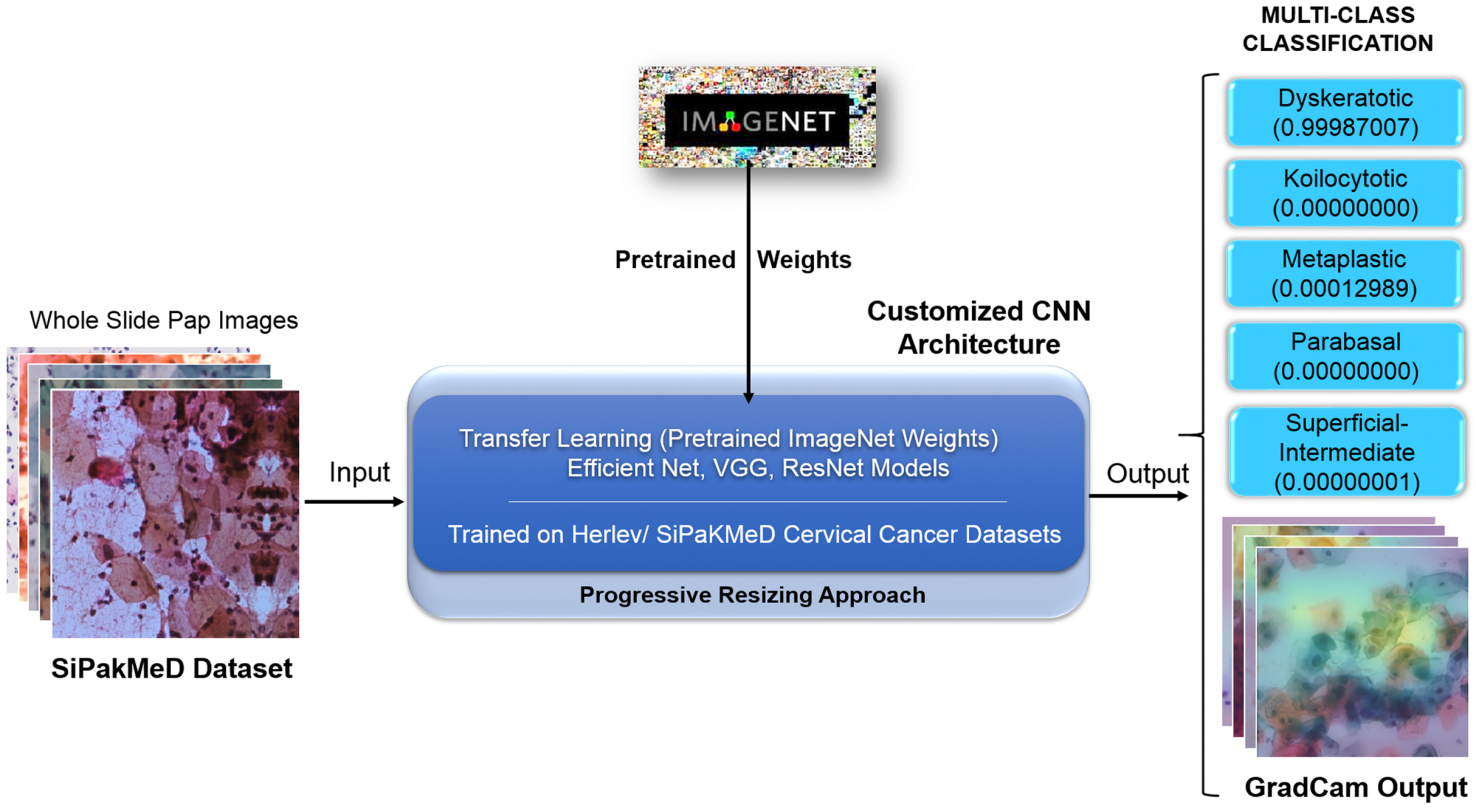
The methodology with inference pipeline used to analyze the Pap smear (Whole Slide) Images using ConvNets (trained using Transfer Learning and Progressive Resizing techniques) to generate Predictions and Activation Map.

We have objected our work to carry out experiments on binary classification for Herlev and SIPakMeD datasets using K-Nearest Neighbour, Stochastic Gradient Descent, Support Vector Machine, and Random Forest classifiers. Then, Convolution Neural Network models, that is, VGG-19 (Simonyan & Zisserman, 2014), ResNet-34 (Szegedy et al., 2016), and EfficientNet-B3 (Tan & Le, 2019) were employed to carry out binary classification where CNNs have shown considerable improvements in the results compared to other classifiers. We carried out due customization of the final layers of CNN models for multi-class classifications for the Herlev and SIPaKMeD datasets. We used pre-trained weights from the ImageNet dataset (referenced as Transfer Learning (Pan & Yang, 2009)) with superimposition of Progressive Resizing techniques while training the CNN models to train Cervical Cancer datasets. Finally, the multi-class classification experiments highlight enhanced results for both datasets. Saliency maps for Whole Slide Images improve assimilation along with classification scores. Figure 5 illustrates the proposed methodology to generate classification results with Saliency Map employing Convolution Neural networks using Transfer Learning and Progressive Resizing techniques. The implementation consists of four stages: (1) Data Preprocessing, (2) Model Implementation, (3) Training Strategy, (4) Testing.

### Data preprocessing

Since data augmentation acts as a regularizer and helps reduce overfitting while training the machine learning model, we used data augmentation techniques to expand the training dataset’s size artificially. We created modified versions of the dataset’s images. We generated skillful models with improved ability to generalize and classify the images by training the deep learning models on more data variations. We implemented transformation techniques to generate cell image variations as the cells are invariant to the rotations. We carried out these transformation with, value of θ = −60 to 60 degrees, α = 1.0 − 1.1, P_A_ = 0.75 and P_B_ = 0.5 while employing the following techniques:-

1. Horizontal flipping of the images with a probability of P_B_.
2. We applied rotations to the images due to their rotational invariance.
3. Random scaling of *α* with a probability of P_A_.

### Model implementation

Herlev and SIPakMeD datasets incorporate single-cell images. Whole Slide Images (WSI) of the SIPaKMeD dataset which expose varied intensity (average intensity, average contrast), texture (smoothness, uniformity, third moment, entropy), and shape features (area, major and minor axis length, eccentricity, orientation, equivalent diameter, solidity, and extent). The proposed CNN implementation deliberate on effective cell morphology feature extraction, that is, cell cytoplasm, cell shape, nucleus type, hyperchromicity, dying exposures of cells, and cell edges. We experimented VGG-19, ResNet-34, ResNet-101, EfficientNet-B3, EfficientNet-B4, and EfficientNet-B5 models. As EfficientNets (image classification models) scale more efficiently by balancing the network’s depth, width, and resolution deliberately over other CNNs, we carried out experiments on EfficientNet models to enhance performance. We have customized the output layers to suitably perform binary and multi-class classification (Tan & Le, 2019). The values of the hyperparameters have been optimized while carrying out experiments. To implement Transfer Learning, we used pre-trained weights obtained after training the model on a large dataset (i.e., ImageNet) while re-training the CNNs on the Herlev and SIPaKMeD datasets. We applied the Progressive Resizing technique by running the iterations repetitively to extract the optimum weights with precise feature extraction. These optimum weights were carried forward to the experiments’ subsequent iteration, where we resized the images from 224 to 256, 512, 1024, and 2048 progressively and ran the experiments. Iterations outcomes were analyzed continuously to achieve the best of the classification scores.

### Training strategy

**Figure 6:**
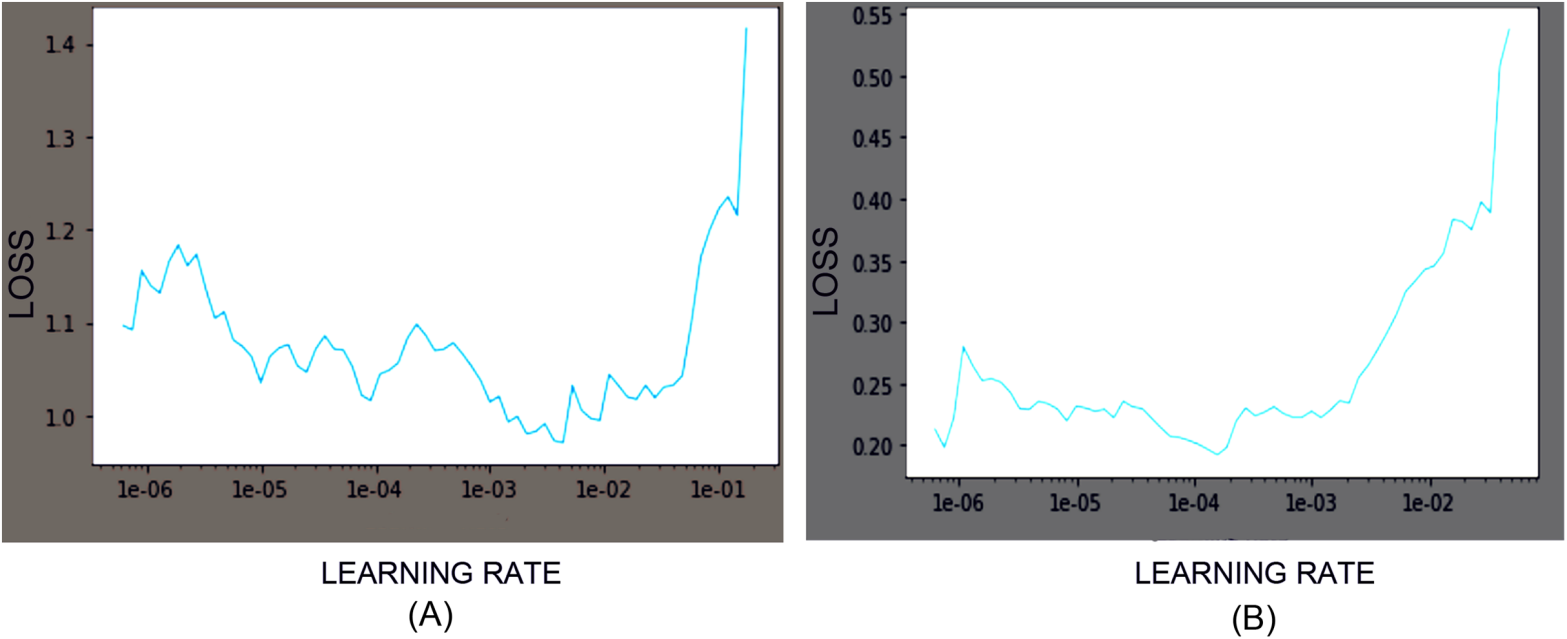
Loss function graphs while training the ResNet-34 CNN model on Herlev Dataset. The learning rate graphs generated after the first iteration (consisting of 30 epoch) and the third iteration (consisting of 30 epochs) using LR Finder are shown at (A) and (B), respectively.

Among the family of EfficientNet models, we used EfficientNet-B3, B4, and B5 CNN models with pre-trained weights obtained from the ImageNet Large Scale Visual Recognition Challenges dataset with 1,000 classes. Models’ customization at the final classification layer was applied to classify the cells into the desired output classes. While training the model for the Herlev dataset and SiPaKMeD dataset, the final classification layers were customized to classify seven and five classes, respectively. Training and test data were split into the 80:20 ratio. Transfer Learning and Progressive Resizing techniques were employed while re-training the CNN models to leverage the ImageNet pre-trained model’s features. We used Categorical Cross—Entropy as the loss function. The learning rates were optimized using the 1-cycle policy, which helped achieve Super-Convergence with faster training, ensuring optimum regularization (Smith & Topin, 2019). We used Weight Decay W_d_ and discriminative learning rates since different layers capture different types of information. It allows us to preserve the filters in the layers closer to input learned by EfficientNet on the ImageNet dataset. The network was trained with AdamW Optimizer (Loshchilov & Hutter, 2017). The learning rates were scheduled using a 1-cycle policy, which enables Super-convergence, allowing faster training of neural networks with substantial learning rates and regularization, preventing overfitting (Yosinski et al., 2014; Smith, 2018). The learning rates were determined using the LR Finder (Howard & Gugger, 2020). Learning Rate was optimized after each epoch to identify the optimal learning rate for the subsequent epoch. Figure 6 illustrates the Learning rate finder’s employment before the first and the third training iterations of the ResNet-34 model on the Herlev dataset.

#### Model customization and experimentation

We carried out binary classification on the Herlev dataset using K-Nearest Neighbour, Stochastic Gradient Descent, Support Vector Machine, and Random Forest classifiers. We applied ResNet-34 and EfficientNet-B3 model to improve the binary classification scores. We used the Transfer Learning technique to train the VGG-19 (baseline), ResNet-34, ResNet-101, EfficientNet B3, EfficientNet B4, and EfficientNet B5 models for multi-class classification on the Herlev dataset. We used discriminative learning rates to preserve the lower level features and regulated the higher-level features for optimum results. The VGG-19 model was trained on both the datasets to carry out binary and multi-class classification as part of the first Conv Net implementation. In VGG-19, convolution layers that analyze the input image features are referred to as “backbone” and balance of the culminating linear layers—referred to as “head.” “Head” converts the analyzed features into predictions for two classes in our binary classification. To train the model with differential learning rates, we split the head from the architecture’s balance. We replaced it with an AdaptiveConcatPool layer, Flatten Layer, and blocks of Batch Normalization, Dropout, Linear, and ReLU layers, respectively. The AdaptiveConcatPool Layer helps preserve the backbone’s feature representations more effectively than using only the MaxPool Layer or the AveragePool Layer. Also, we appended a fully-connected layer of 7 and 5 units with softmax activation as Final Classification Layer for Herlev and SIPaKMeD datasets, respectively. We carried out suitable customization of all the Conv Net for respective datasets, which attained improved performance scores. Hyperparameters—the properties that govern the entire training process and determines the network structure were carefully optimized. Learning Rate was optimized after each epoch to identify the optimal learning rate for the subsequent epoch. We determined the number of Epochs and Activations Functions, considering numerous experimental results, and used suitable optimized values while carrying out experiments.

**Figure 7:**
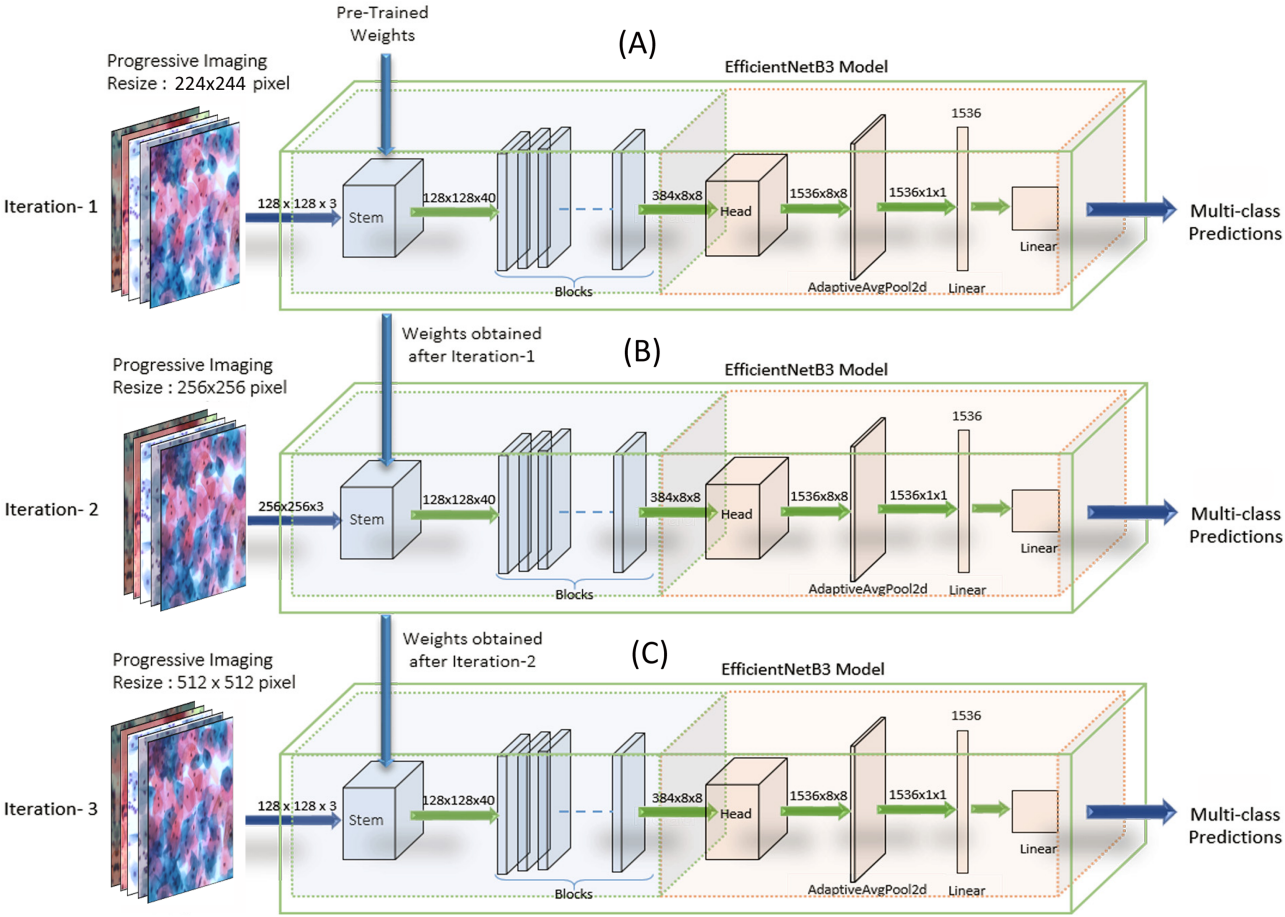
The implementation methodology for the Progressive Resizing Technique with Transfer Learning using the EfficientNet CNN model. Pre-trained weights obtained by training the model on large datasets are taken as initializing weights on the model (A) (i.e., Imaging input size of 224 × 224 pixels initially), and then carry forward the obtained weights to subsequent models (B) and (C), (i.e., Imaging input size to 256 × 256 pixels and 512 × 512 pixels respectively) to extract optimum features and enhanced efficiency progressively.

EfficientNets—image classification models scale more efficiently by deliberate balancing the network’s depth, width, and resolution. Hence, the models have demonstrated enhanced performance. We have customized the output layers suitably for binary and multi-class classification (Tan & Le, 2019) on both the datasets. We carried out experiments on EfficientNet B0, B1, B2, B3, B4, and B5. Though the EfficientNet higher variants, that is, B4-B7, have larger width, depth, or resolution, the accuracy gain was saturating/stable compared to the B3 model, which demonstrated the limitation of a single dimension scaling. Hence, experiments were carried out on EfficientNet B1, EfficientNet B2, and EfficientNet B3. We used the baseline EfficientNet-B3 model for binary and multi-class classification on our Cervical cancer datasets. For WSI Image analysis, we used the Progressive resizing technique (Howard & Gugger, 2020) on these convolutions Neural Network models. We trained the model with Imaging, sized 224 × 224, to obtain the weights. Then using these weights, we trained the model with resized the WSI images and iterated the model’s training repetitively by gradually increasing Imaging sizes to 256 × 256, 512 × 512, 1024 × 1024, followed by unfreezing saved weights (from the previous iteration) every time as each larger-scale model incorporates the previous smaller-scale model layers and weights. We observed significant results by following the Strategy. Figure 7 shows the implementation methodology of Progressive resizing on the Whole Slide Images by applying the obtained weights from one to the next training model by scaled-up image resizing.

### Testing

During the testing, we resized the input image to (H × W) size and gave the resized images as input to the networks to predict the output class. For all the tasks on the SIPaKMeD dataset, we used 3-fold and 5-fold cross-validations with the data split released along with the dataset.

## Results and Discussion

### Evaluation parameters

As the accuracy in isolation does not prove the model’s efficacy, we have evaluated the model based on the performance metrics. The performance of the model is ascertained post-study of the scores, that is, Accuracy (Acc), Precision, Sensitivity (Sens), Specificity (Spec), H-Mean, *F*1-score, or F-Beta, and Kappa Score followed by an independent manual analysis by the Pathologists. Sensitivity or recall measures the proportion of actual abnormal cells that are predicted correctly in the same class. Specificity measures the proportion of actual negatives and correctly identified as such. Accuracy is the overall percentage of correctly identifying the cells belonging to the respective classes correctly. *F*1 score is a classifier metric that calculates a mean of precision and recall.

$$\rm Accuracy = \frac{TP+TF}{TP+TF+FP+FN}$$

$$\rm Precision = \frac{TP}{TP+FP}$$

$$\rm Recall = \rm\frac{TP}{TP+FN}$$

$$ F1{\rm } = {\rm }\displaystyle{{\rm 2*Precision*Recall} \over {\rm Precision + Recall}}{\rm } = {\rm }\displaystyle{\rm{2*TP} \over {\rm 2*TP + FP + FN}}$$

We used Cohen’s Kappa Score (Cohen, 1960, 1968), which is a statistical measure for measuring “Intra-rater Reliability” and is a more robust measure than simple percent agreement calculation. It is a measure of agreement between observations and can be defined for two observers and where disagreement was weighted without taking into account the distance between categories. The weighted kappa statistic κw for two observers and ensures that there is no weighted kappa statistic for more than two observers. We have used the following notation is used:

- *N* the number of cases/observations.
- *n* the numbers of raters/observers.
- *k* the number of categories in which a case can be rated.

Cohens weighted kappa *κ*_*w*_ is derived from the normal kappa by including a weight function *w*. If *w* is chosen the identity matrix *I*_*k*_, then Cohens *κ*_*w*_ is identical to Cohens *κ*. A linear weight is commonly chosen, which is calculated as:

(1) $${w_{ij}} = 1 - \displaystyle{{|i - j|} \over {k - 1}}.$$

Alternatively, a quadratic weight could be used: ${w_{ij}} = 1 - {(i - j)^2}/(k - {1)^2}$. Then:

(2) $${P_{o(w)}} = \displaystyle{1 \over N}\sum\limits_{i = 1}^k \sum\limits_{j = 1}^k {w_{ij}}{f_{ij}},$$

(3) $${P_{e(w)}} = \displaystyle{1 \over {{N^2}}}\sum\limits_{i = 1}^k \sum\limits_{j = 1}^k {w_{ij}}{r_i}{c_j},$$

with *r*_*i*_ and *c*_*j*_ again the row and column sums. The weighted kappa statistic is now given by:

(4) $${\kappa _w} = \displaystyle{{{P_{o(w)}} - {P_{e(w)}}} \over {1 - {P_{e(w)}}}}.$$

### Quantitative results

**Figure 8:**
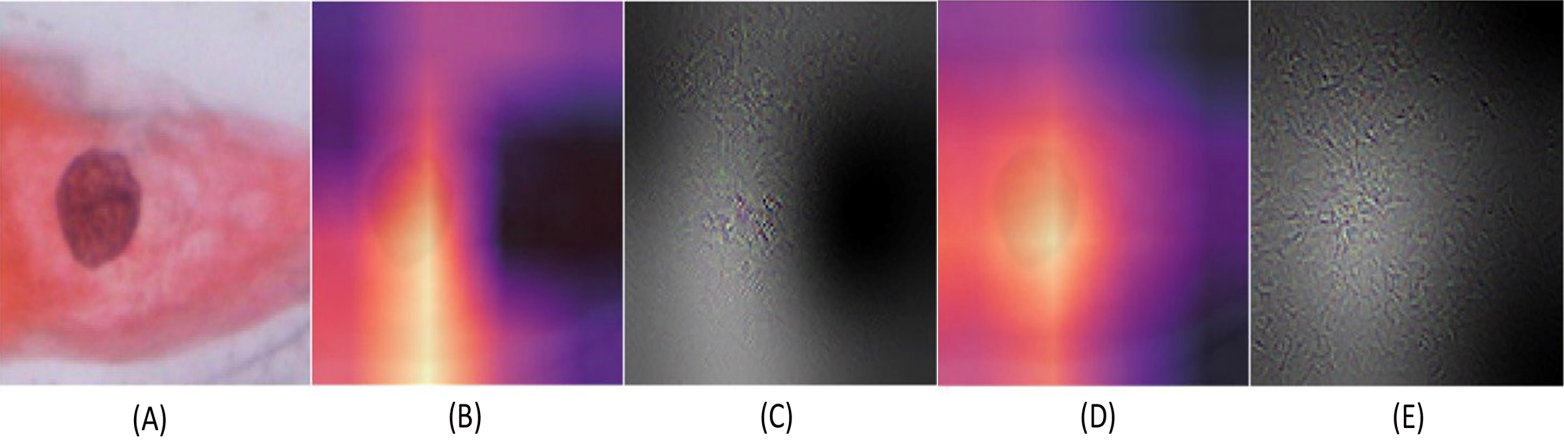
The Binary classification predictions generated using Resnet-101 (trained on Herlev dataset) and Resnet-34 (trained on SIPaKMeD dataset) for a single-cell image. The Resnet-101 and Resnet-34 CNN generated predictions with 99.92% and 98.94% for an abnormal single-cell image input (shown at (A)). The GradCam visualization and CNN feature interpretation, obtained using Resnet-101, are shown at (B) and (C), and Resnet-34 are shown at (D) and (E), respectively.

**Figure 9:**
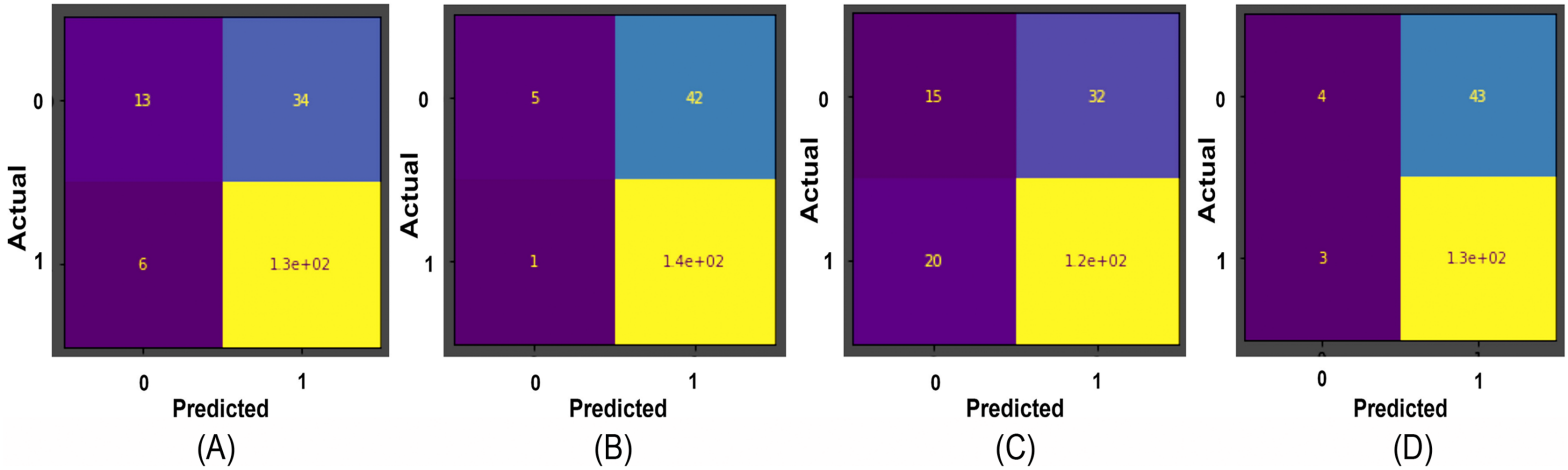
The confusion matrices for binary classification predictions at (A) Support Vector Machines, (B) Stochastic Gradient Descent, (C) K-Nearest Neighbour, and (D) Random Forest Confusion Matrices on the Herlev dataset.

Results obtained for binary classification using K-Nearest Neighbour, Stochastic Gradient Descent, Support Vector Machine, Random Forest classifier, ResNet-34, and EfficientNet-B3 models on Herlev and SiPaKMeD datasets are presented in this section. For the Herlev dataset, the ResNet-34 and EfficientNet-B3 models produced benchmark binary classification scores with 98.91% accuracy, 99.29% precision, 98.92% recall, 98.91% specificity, and 99.10%F1-Score and 99.01% accuracy, 99.15% precision, 98.89% recall, 99.02% specificity, and 98.87% F-Beta score. Table 1 illustrates the quantitative comparisons of binary classification results (Herlev dataset) obtained by experimenting with K-Nearest Neighbour, Stochastic Gradient Descent, Support Vector Machine, and Random Forest classifier, ResNet-34, and EfficientNet-B3 models. Figure 8 shows the binary classification of an abnormal single-cell image using Resnet-101 (trained on Herlev dataset) and Resnet-34 (trained on SIPaKMeD dataset) CNNs. The results of the SiPaKMeD dataset are also shown in the table. Figure 9 shows the confusion matrices generated using K-Nearest Neighbour, Support Vector Machine, Stochastic Gradient Descent, Random Forest for binary classification on the Herlev dataset.

**Table 1:** The binary classification prediction scores for Herlev and SiPaKMeD Cervical Cancer datasets under evaluation criteria, that is, Accuracy, Precision, Recall, *F*1-Score, and Kappa Score. The table demonstrates the scores for various binary classifiers and CNN models, that is, K-Nearest Neighbour, Support Vector Machine, Stochastic Gradient Descent, Random Forest, ResNet-34 (Baseline), and EfficientNet-B3 (Baseline).

Model	Accuracy (%)	Precision (%)	Recall (%)	*F*1-Score (%)	*K*-Score (%)
**Herlev Dataset**					
K-Nearest Neighbour	78.142	79.268	95.588	86.666	–
Support Vector Machine	76.502	76.271	99.264	86.261	–
Stochastic Gradient Descent	71.584	78.378	85.294	81.690	–
Random Forest	74.863	75.568	97.794	85.256	–
**SIPaKMeD Dataset**					
ResNet-34	98.919	99.291	98.925	98.918	99.103
EfficientNet-B3	99.011	99.157	98.896	99.026	98.879

**Figure 10:**
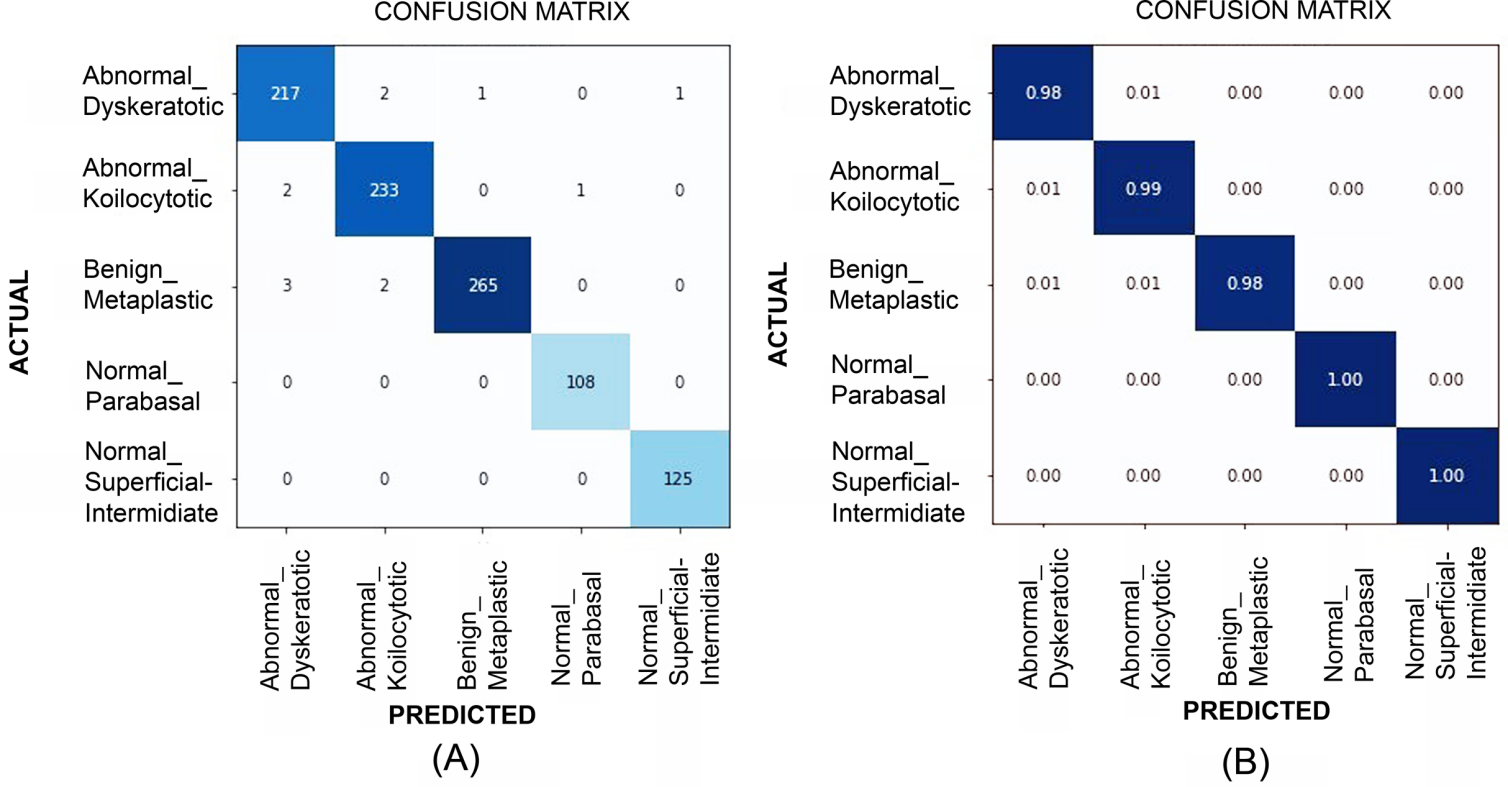
The confusion matrices at (A) and generalized score at (B) for multi-class classification results (for SiPaKMeD dataset) obtained with the EfficientNet-B3 model. The benchmark classification score highlights the optimum results achieved by employing Transfer Learning in consultation with Progressive Resizing.

Table 2 illustrates the quantitative comparison of multi-class classification results (Herlev dataset) obtained by experimenting with VGG-19, ResNet-34, ResNet-101, EfficientNet B2, EfficientNet B3, EfficientNet B4, and EfficientNet B5 models. Multi-class classification scores on SiPaKMeD dataset obtained by implementing VGG-19, ResNet-34, ResNet-101, and EfficientNet-B3 (baseline) using transfer learning viz-a-viz of EfficientNet-B3 using transfer learning, and Progressive resizing technique are also shown in the Table 2. The ResNet-101 (Baseline) and EfficientNetB3 (512 × 512) with progressive image resizing attained the highest scores. Implementations with the proposed technique have attained perfect scores in Sensitivity, Specificity, and the best Kappa Scores. F1-scores of these models indicate the robustness in multi-class classification. EfficientNet B3 models have been evaluated against all possibilities of overfitting. The multi-class classification results demonstrated improved feature extraction and computation efficiency with progressive resizing. Figure 10 shows the confusion matrices of the multi-class classification generated using EfficientNet-B3 CNN on the SIPaKMeD dataset. We did experiments on EfficientNet B3 with pre-trained weights and training them on 224 × 224, 256 × 256, 512 × 512, and 1024 × 1024 progressively. The 5-fold cross-validation indicates that the model did not overfit.

**Table 2:** The multi-class prediction scores for the Herlev and SIPaKMeD Cervical Cancer datasets under evaluation criteria, that is, Accuracy, Precision, Recall, F-Beta, and Kappa Score. Multi-class classification Convolution Neural Network used the K-fold cross-validation techniques, which showed a substantial increase in scores. The EfficientNet-B3 in consultation with Transfer Learning and Progressive resizing generated Benchmark scores for Whole-Slide images of the SIPakMeD dataset.

Model	K-Fold CV	Accuracy (%)	Precision (%)	Recall (%)	F-Beta (%)	Kappa Score (%)
**Herlev**						
VGG19	5-Fold	85.18 ± 6.90	88.22 ± 4.66	87.24 ± 7.00	87.20 ± 6.76	91.26 ± 5.40
ResNet-34	5-Fold	91.94 ± 8.73	93.36 ± 7.41	92.81 ± 8.43	92.86 ± 8.28	95.31 ± 5.05
ResNet-101	5-Fold	93.14 ± 8.78	94.56 ± 7.01	93.98 ± 8.08	94.05 ± 7.93	95.57 ± 5.55
EfficientNetB3	5-Fold	91.40 ± 10.25	92.74 ± 8.63	92.20 ± 9.36	92.19 ± 9.36	92.99 ± 8.04
EfficientNetB4	5-Fold	93.03 ± 8.95	94.31 ± 7.31	94.27 ± 7.62	94.25 ± 7.58	96.40 ± 4.51
EfficientNetB5	5-Fold	92.16 ± 9.25	93.19 ± 9.13	93.21 ± 8.23	93.12 ± 8.28	95.29 ± 4.63
**SIPaKMeD**						
VGG19	5-Fold	98.65 ± 0.57	98.79 ± 0.64	98.44 ± 0.48	98.90 ± 0.48	99.09 ± 0.41
ResNet-34	3-Fold	96.56 ± 0.13	97.19 ± 0.29	97.27 ± 0.17	97.22 ± 0.14	98.19 ± 0.13
ResNet-101	5-Fold	98.55 ± 1.11	98.57 ± 1.26	98.70 ± 0.88	98.65 ± 0.97	98.07 ± 1.47
EfficientNetB3	3-Fold	97.81 ± 0.10	98.09 ± 0.10	98.23 ± 0.23	98.24 ± 0.21	98.58 ± 0.15
EfficientNetB3	5-Fold	99.27 ± 0.74	99.36 ± 0.69	99.43 ± 0.61	99.41 ± 0.63	99.54 ± 0.60
EfficientNetB3	5-Fold	96.39 ± 1.85	96.49 ± 1.42	97.02 ± 1.59	96.88 ± 1.56	97.37 ± 1.12
EfficientNetB3	5-Fold	99.70 ± 1.07	99.70 ± 1.03	99.72 ± 0.87	99.63 ± 0.88	99.31 ± 0.78

### Results validation

We used the annotated Herlev and SIPaKMeD datasets. However, the data were analyzed and reviewed by an expert pathologist in a secondary care hospital. An extensive set of Whole Slide Images were shown to the experts for manual analysis and inspection to acquaint the dataset. We carried out validation tests to ascertain the results generated by the system. The validation was carried out by manual Kappa Score tally. 35 × Whole Slide Images were segregated for the validation test, which was not part of training/testing datasets. Independent pathologists and the trained model were given 35 × WSI images one by one for analysis. The system prediction scores were tallied with expert analysis. Out of 35 × WSI images, 34 × WSI results matched. The expert could not interpret one WSI image due to low image quality. Observer bias was ruled out for each diagnosis, under the same microscopic setup and view by taking in the review of two more pathologists to authenticate diagnosis by the system.

### Saliency maps

**Figure 11:**
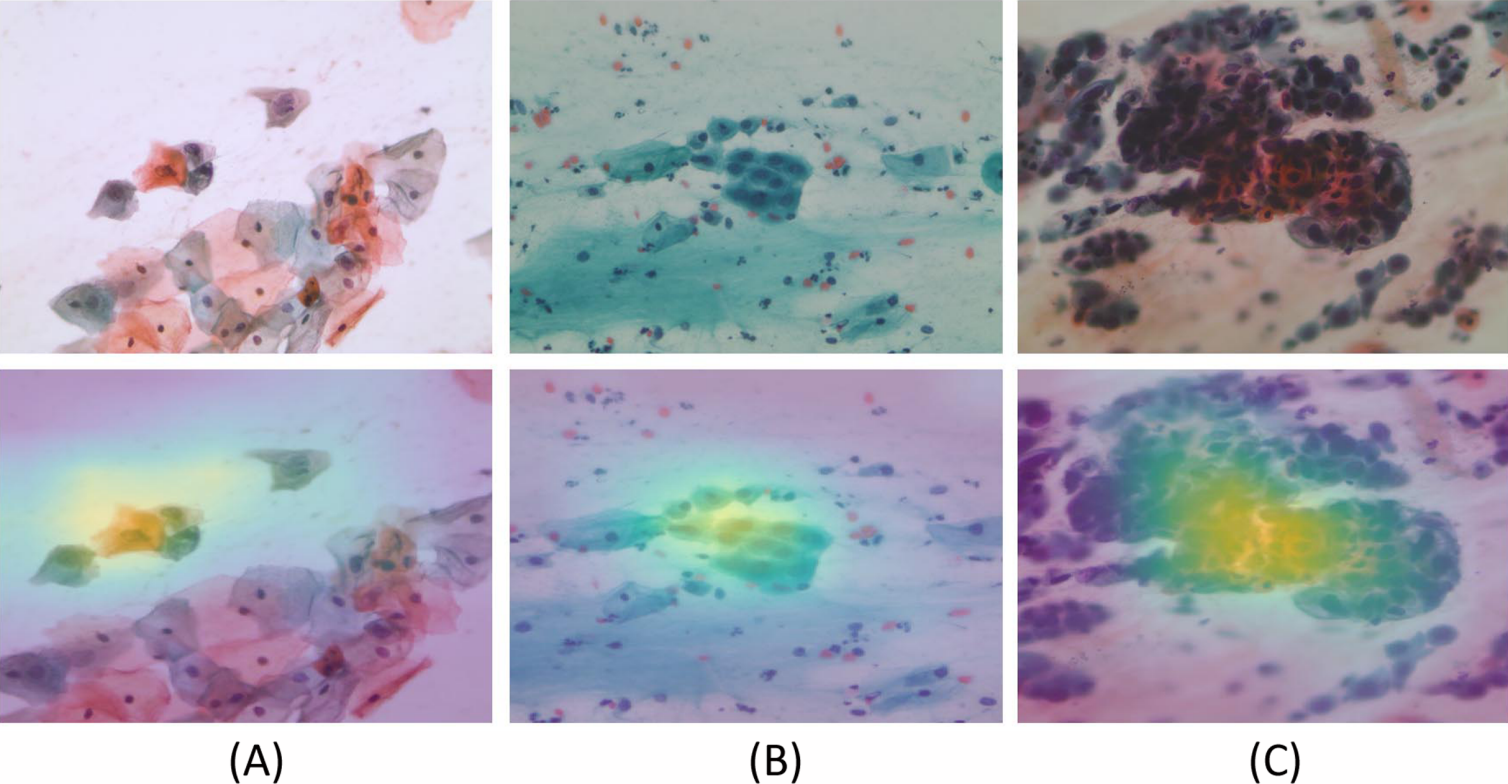
The model generated Gradient-weighted Class Activation Maps for the input Pap smear images, respectively. Input Pap smear and generated Gradient-weighted Class Activation Maps show results for (A) Abnormal ‘Koilocytotic,’ (B) Benign ‘Metaplastic,’ and (C) Abnormal ‘Dyskeratotic’ classes.

We generated Grad-CAM heat-maps using the final Layer of a Convolution Neural Network. To assimilate the results generated by the system and enhanced manual validation, Saliency Maps, that is, Gradient-weighted Class Activation Map (GradCAM) was implemented (Selvaraju et al., 2017). In Adaptive Average Pooling, we took the average of all the 1 × 1 × 512 channels. We converted them to a tensor of 512, followed by multiplying the tensor with size (512 × no. of classes) to get the final output. While validating multi-class experiments for whole slide Pap smear images, we took five classes. The 512 values represented the features extracted by the convolutions layers, which are matrices. We took the first cell’s average across every channel to show activated areas when the model predicted a specific class. To generate the heatmaps, we used “hooks.” Figure 11 shows the Grad-CAM for various class inputs by highlighting the feature extraction and predictions for abnormal and benign WSI classes.

## Conclusions

In this research, we proposed a binary and multi-class classification pipeline for identifying carcinoma in Pap smear images to carry out metamorphic diagnosis of neoplastic and pre-neoplastic lesions. We are inspired by the emerging role of CNNs to aid medical imaging-based diagnostics, which can play as a digital second opinion to improve palliative care. The proposed pipeline starts with a cervical cell recognition stage. To begin with, we carried out experiments with binary classifiers and obtained performance scores. Towards the further enhancements, we improved ResNet-34, ResNet-101, and EfficientNet B3 models’ performance with Transfer Learning. The ResNet-34 and EfficientNet B3 trained with the Transfer Learning technique showed a significant increase in performance, achieving 98.91% accuracy, 99.29% precision, 98.92% recall, 98.91% specificity, 99.10% *F*1-Score, and 99.01% accuracy, 99.15% precision, 98.89% recall, 99.02% specificity, the 98.87% F-Beta score for SIPaKMeD Dataset, respectively. In a later stage, we carried out multi-class classification experiments on both datasets. We addressed a problem that has not been addressed effectively by any literature, that is, Whole Slide Image analysis with multi-class classification. We proposed a novel approach of implementing Progressive Resizing and Transfer Learning on Deep Neural Network models, which attained State-of-the-Art computational efficiency and best of the scores for accurate classifications. Convolution Neural Network model- EfficientNet-B3 trained using Transfer Learning, and Progressive Resizing showed promising multi-class classification results. We achieved benchmark scores on WSI by effectively analyzing multi-layer cervical cells with, that is, 99.70% Accuracy, 99.70% Precision, 99.72% Recall, 99.63% Specificity, and 99.31% F-Beta score. We outperformed other techniques cited in recent literature, consistently in both types of classification problems. We ascertained the system generated predictions by a validation test where an expert’s manual analysis was tallied with prediction. We also showed the model’s transparency by visualizing the features learned by saliency maps, which enabled us to visualize the CNN generated predictions.

## Supplemental Information

10.7717/peerj-cs.348/supp-1 Supplemental Information 1 Code Herlev Dataset Binary Classification.

10.7717/peerj-cs.348/supp-2 Supplemental Information 2 Code Herlev Dataset Multiclass assification EfficientNetB5.

10.7717/peerj-cs.348/supp-3 Supplemental Information 3 Code Herlev Dataset Multiclass Classification EfficientNetB4.

10.7717/peerj-cs.348/supp-4 Supplemental Information 4 Code Herlev Dataset Binary Classification EfficientNet B3.

10.7717/peerj-cs.348/supp-5 Supplemental Information 5 Code Herlev Dataset Multiclass Classification VGG-19.

10.7717/peerj-cs.348/supp-6 Supplemental Information 6 Code Herlev Dataset Multiclass Classification ResNet101.

10.7717/peerj-cs.348/supp-7 Supplemental Information 7 Code Herlev Dataset Multiclass Classification resNet34.

10.7717/peerj-cs.348/supp-8 Supplemental Information 8 Code Herlev Dataset Multiclass Classification ResNet34 5fold CV.

10.7717/peerj-cs.348/supp-9 Supplemental Information 9 Code SIPAKMED Dataset Multiclass Classification VGG-19.

10.7717/peerj-cs.348/supp-10 Supplemental Information 10 Code SIPAKMED Dataset Multiclass Classification resnet101.

10.7717/peerj-cs.348/supp-11 Supplemental Information 11 Code SIPAKMED Dataset Multiclass Classification Progressiive Resizing.

10.7717/peerj-cs.348/supp-12 Supplemental Information 12 Code SIPAKMED Dataset Multiclass ClassificationEff Net B3.

10.7717/peerj-cs.348/supp-13 Supplemental Information 13 Code SIPAKMED Dataset Multiclass Classification VGG-19.

10.7717/peerj-cs.348/supp-14 Supplemental Information 14 Code SIPAKMED Dataset Multiclass ClassificationResNet 34 3 fold CV.

10.7717/peerj-cs.348/supp-15 Supplemental Information 15 Code SIPAKMED Dataset Multiclass Classification ResNet101.

10.7717/peerj-cs.348/supp-16 Supplemental Information 16 Code SIPAKMED Dataset Multiclass Classification EffNetB3 3 Fold.

10.7717/peerj-cs.348/supp-17 Supplemental Information 17 Code SIPAKMED Dataset Multiclass Classification EffNet B3 Baseline.

10.7717/peerj-cs.348/supp-18 Supplemental Information 18 Code SIPAKMED Dataset Multiclass Classification EffNet B3 5Fold.

## Additional Information and Declarations

### Data Availability

The following information was supplied regarding data availability: Source code is available in the Supplemental Files. Data are available at the Herlev Cervical Cancer Database (http://mde-lab.aegean.gr/index.php/downloads; specifically Part II: "New Pap-smear Database (images)") and the SIPaKMeD Cervical Cancer Database (https://www.cs.uoi.gr/~marina/sipakmed.html; data from all cell categories were used).
